# Magnetic resonance imaging reveals possible cause of diplopia after Baerveldt glaucoma implantation

**DOI:** 10.1371/journal.pone.0276527

**Published:** 2022-10-20

**Authors:** Esma Islamaj, Luc Van Vught, Caroline P. Jordaan-Kuip, Koenraad A. Vermeer, Teresa A. Ferreira, Peter W. T. De Waard, Hans G. Lemij, Jan-Willem M. Beenakker

**Affiliations:** 1 Rotterdam Ophthalmic Institute, Rotterdam Eye Hospital, Rotterdam, Netherlands; 2 Department of Ophthalmology, Leiden University Medical Center, Leiden, Netherlands; 3 Department of Radiology, Leiden University Medical Center, Leiden, Netherlands; 4 Department of Glaucoma, Rotterdam Eye Hospital, Rotterdam, Netherlands; Cairo University Kasr Alainy Faculty of Medicine, EGYPT

## Abstract

**Purpose:**

To assess if ocular motility impairment, and the ensuing diplopia, after Baerveldt Glaucoma device (BGI) implantation, is related to the presence of a large fluid reservoir (bleb), using Magnetic Resonance Imaging (MRI).

**Methods:**

In a masked observational study (CCMO-registry number: NL65633.058.18), the eyes of 30 glaucoma patients with (n = 12) or without diplopia (n = 18) who had previously undergone BGI implantation were scanned with a 7 Tesla MRI-scanner. The substructures of the BGI-complex, including both blebs and plate, were segmented in 3D. Primary outcomes were a comparison of volume and height of the BGI-complex between patients with and without diplopia. Comparisons were performed by using an unpaired t-test, Fisher’s Exact or Mann-Whitney test. Correlations were determined by using Spearman correlation.

**Results:**

The median volume and height of the BGI-complex was significantly higher in patients with compared to patients without diplopia (p = 0.007 and p = 0.025, respectively). Six patients had an excessively large total bleb volume (median of 1736.5mm^3^, interquartile range 1486.3–1933.9mm^3^), four of whom experienced diplopia (33% of the diplopia patients). Fibrotic strands through the BGI plate, intended to limit the height of the bleb, could be visualized but were not related to diplopia (75% versus 88%; p = 0.28).

**Conclusions:**

With MRI, we show that in a significant number of diplopia cases a large bleb is present in the orbit. Given the large volume of these blebs, they are a likely explanation of the development of diplopia in at least some of the patients with diplopia after BGI implantation. Additionally, the MR-images confirm the presence of fibrotic strands. As these strands are also visible in patients with a large bleb, they are apparently not sufficient to restrict the bleb height.

## Introduction

As vision lost due to glaucoma cannot be recovered, glaucoma treatment aims at preventing (further) loss. At present, lowering the intraocular pressure (IOP) is the only proven preventive method^1^. When treatment with topical medication or laser therapy is not effective in lowering the IOP, the ophthalmologist may opt for incisional surgery. A commonly used surgical method is the implantation of a Baerveldt glaucoma drainage implant (BGI) in the orbit, adjacent to the eye [1]. This implant is non-valved and contains a small open unobstructed drain, commonly placed in the anterior chamber. During surgery the drain is tied off with a single 7.0 vicryl suture, which dissolves after 4–6 weeks, allowing the drain to extract a part of the aqueous humour flows, thus lowering the IOP. The drain is connected to a plate, typically positioned under the rectus muscles, which, by its encapsulation, serves as a temporary reservoir. The silicone plate itself features 4 fenestrations, which are intended for the growth of fibrotic strands during encapsulation. After encapsulation, the silicone plate is mostly surrounded by one or two fluid compartments, further referred to as blebs. The strands keep the blebs, surrounding the plate, flat, with the intention to limit restrictions in eye motility, caused by the bleb.

Although the BGI has shown success in controlling the IOP [2–4], some persistent postoperative complications may occur. After BGI surgery, 5–28% of the patients suffer from double vision, also called diplopia [5–9]. Diplopia may be a severe complication that needs proper attention as it has a negative impact on the quality of life. The most common issues for patients with diplopia are *impaired reading*, impaired *driving*, ne*gative feelings in self-esteem, and general disability [10]*. In addition, some patients develop a torticollis to maintain binocular single vision, resulting in neckpain [11, 12].

Diplopia, after BGI surgery, is most likely caused by ocular motility impairment [7, 8], but it is not fully understood how the BGI plate restricts eye movement. Due to its location in the orbit, it is difficult to visualize the BGI, and the surrounding anatomy, in a non-invasive way, as optical imaging techniques, including fundoscopy and OCT lack the penetration depth to assess retrobulbar structures. Although it is possible to image the BGI with ultrasound, it is unable to provide an accurate 3D visualisation as the resolution will always be low in one direction [13]. Additionally, ultrasound images often do not visualise the complete implant and the contrast is too low to detect the fibrotic encapsulation of the BGI.

Previous studies have shown how ocular magnetic resonance imaging (MRI) is able to visualize the BGI and its surrounding structures in glaucoma patients [14–16]. However, to our knowledge, MRI has never been combined with orthoptic measurements to uncover the cause of diplopia after glaucoma surgery. We hypothesized that, after BGI implantation, the developed bleb size is correlated to the presence of any ocular motility impairment, and the ensuing diplopia. This hypothesis was tested by using high-field MRI.

## Methods

### Patient selection

This masked observational study was approved by the medical ethics committee of the Leiden University Medical Center (LUMC) (Leiden, the Netherlands, CCMO-registry number: NL65633.058.18). The study protocol adhered to the tenets of the Declaration of Helsinki. Patients were retrospectively selected from previous studies in which diplopia and motility impairment was investigated until 1 year after BGI surgery [8, 17]. In- and exclusion criteria of these studies are previously described in detail [8, 17]. Patients were excluded when monocular diplopia was present, they had a history of strabismus or were monocular. Patients were subsequently divided into 2 groups based on the presence or absence of diplopia at the 1-year postoperative assessment. The patients’ medical ocular history was obtained from their medical records. Before participation, the patients were screened for contraindications for MRI scanning. Study measurements were only performed after obtaining written informed consent.

### Surgical procedures

Two surgeons (H.L. and P.d.W.) had performed all BGI surgeries at the Rotterdam Eye Hospital using a commercially available BGI (BG-101-350, Abbott Medical Optics (Johnson & Johnson Surgical Vision, Inc.)). The BGI tube was inserted into the anterior chamber through a sclerostomy. The sclerostomy was made in such a way that the tube entered the sclera approximately 2 mm posterior to the limbus. The BGI plate was placed in the superotemporal or superonasal quadrant, with its wings placed under the rectus muscles. The plate was sutured or placed freely on the sclera, depending on the technique of the surgeon. By not suturing the plate to the globe, i.e. placing the plate ‘freely’, it was thought that it would find a position in which the plate and its encompassing bleb would minimally restrict normal eye movements.

### Orthoptic measurements

Orthoptic measurements were obtained by an orthoptist. To quantify diplopia, we asked patients if they experienced any diplopia. Eight different ductions were measured by means of a Goldmann perimeter (Haag Streit, Köniz, Zwitserland), including elevation, depression, adduction, abduction, elevation in abduction, elevation in adduction, depression in abduction and depression in adduction. To measure ductions using the Goldmann perimeter we followed Gerling’s method [18], The fellow eye was occluded and the strongest spotlight was used (relative intensity 4e [1.0], object V [64 mm^2^]). A significantly restricted duction measured was defined as a loss of at least 10° of duction compared to the duction range of a normal adult eye [19]. The threshold of 10° was based on the repeatability of motility measurements and age-related restrictions [19, 20]. The latter refers to the possible gradual decrease in both horizontal and vertical gaze directions with advancing age, especially around the sixth decade. Upon the presence of multiple restrictions in different gaze directions (e.g. in elevation, depression, etc.), all restrictions were included in the analyses. Finally, the visual field (VF) of the study eye of the patient was considered. The patient’s mean deviation (MD) was classified into 3 visual field loss categories after Hodapp-Parrish-Anderson; mild (MD>-6dB), moderate (-6dB>MD>-12dB) or severe (MD<-12dB) [21].

### Magnetic resonance imaging

All patients were scanned with a 7 Tesla Philips Achieva MRI (Philips Healthcare, Best, The Netherlands) by using the anterior 16 channels of a 32-channel head-coil (Nova Medical, Wilmington, MA, USA) at the LUMC. A cued-blinking paradigm was used to reduce any motion artefacts by controlling ocular motion [22]. A three-dimensional T2-weighted (3DT2) scan with an isotropic acquisition resolution of 0.8 mm was acquired for each subject to assess the 3D geometry of the BGI and bleb. The 3DT2 scan used a turbo-spin-echo readout with an effective echo time of 191.5 ms, a repetition time of 2500 ms, a refocusing angle of 35 degrees, an echo train length of 120, and a reconstruction resolution of 0.4 x 0.4 x 0.4 mm^3^. The scan covered both orbits with an acquisition time of just over 2 minutes. Additionally, two-dimensional T2-weighted (2DT2) images were acquired with an increased in-plane resolution for a more detailed assessment of the interface between the bleb and surrounding structures. This 2DT2 scan was acquired with an in-plane resolution of 0.7x 0.7 mm^2^, reconstructed to 0.35mm x 0.35mm, a slice thickness of 1.5 mm, an echo time of 63 ms, and a repetition time of 3400 ms. The scan duration of the 2DT2 was 1 minute and 42 seconds. Two sets were acquired, one planned perpendicular to the outer bleb of the BGI and one planned perpendicular to the two rectus muscles that were closest to the BGI.

All MR-images were screened by a neuro-, head and neck radiologist (T.F.) for any other visible pathology and scored by an experienced ophthalmic MRI specialist (J.W.B.) for the presence of fibrotic strands and atypical aspects of the structures surrounding the BGI. Both, radiologist and MRI specialist were masked to the presence/absence of diplopia during this study.

### Quantification of the BGI volume and height

**Fig 1:**
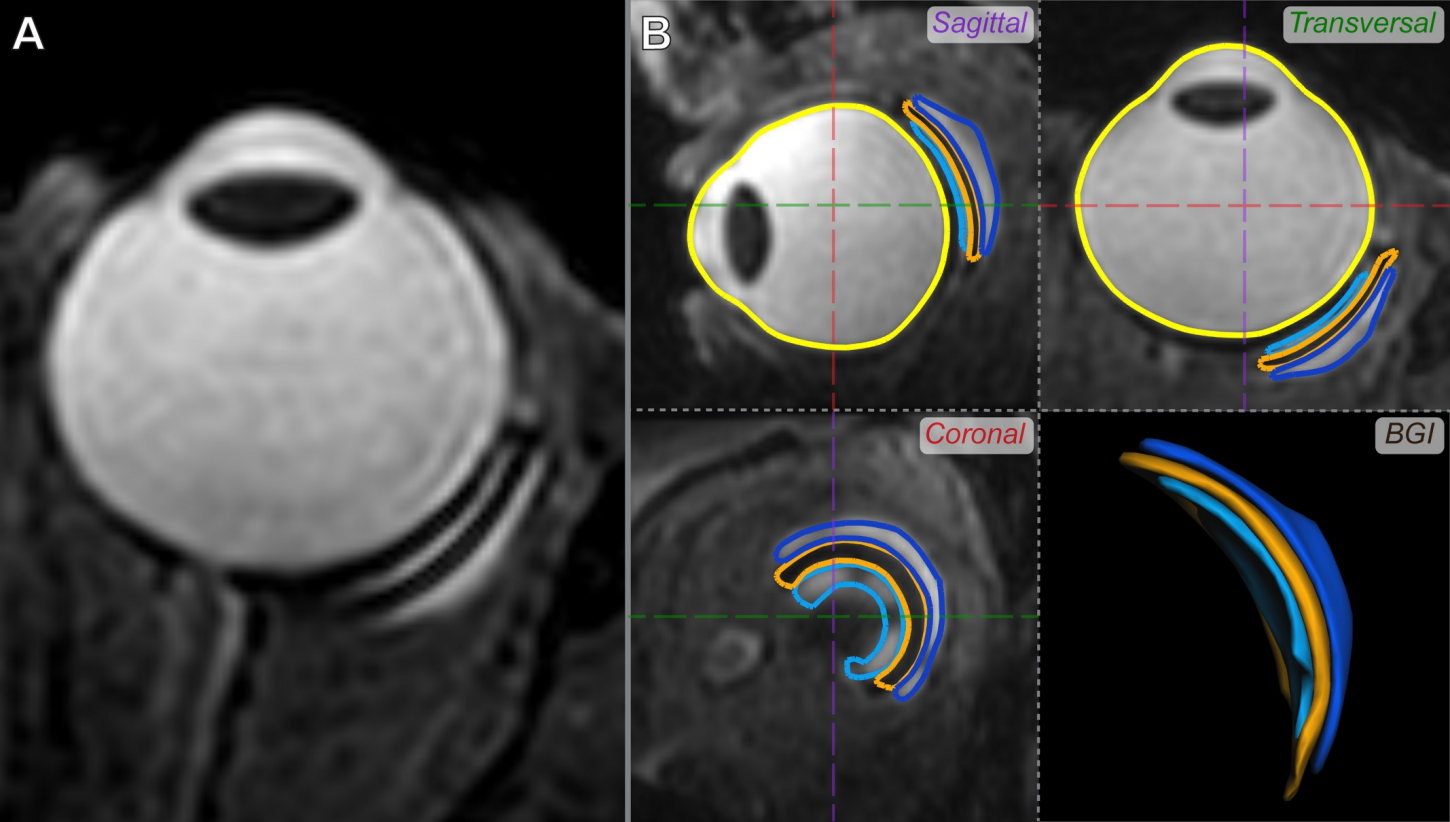
Three-dimensional MRI assessment of the BIG. A) Typical 3D T2-weighted MR-image of an eye with a BGI. B) Volumes segmented from that same MR-Image in three orthogonal reformats (sagittal, transversal and coronal), allowing for an accurate and 3D assessment of the bleb and its relation to the eye. Displayed are the inner bleb *(dark blue)*, BGI *(orange)* and outer bleb *(light blue)*. A segmentation of the eye *(yellow)* is also shown for reference.

The 3D volume and height of the BGI were obtained by segmenting the inner bleb, outer bleb and plate of the BGI 3DT2 MR-images, later referred to as the BGI complex. To this end, a semi-automated segmentation of these structures was performed in ITK-snap [23], which was used as input for subsequent, fully automated boundary detection by means of subdivision fitting (Fig 1B) in MeVisLab (version 3.0.2, MeVis Medical Solutions AG, Bremen, Germany). All segmentations were checked by an experienced reader (J.W.B.). The resulting 3D meshes were voxelized to an isotropic resolution of 0.1 mm^3^ and small gaps between the individual segmentations were corrected by using a cascade of morphological operations if applicable, providing a volume for each individual component. The height of the total BGI complex was subsequently calculated as the largest distance between the outer surfaces of the inner and outer bleb, perpendicular to the plate.

### Primary outcomes

Primary outcomes of this study were the volume and height of the BGI complex, including both its fluid reservoirs (blebs) and the plate, compared between patients with and without diplopia.

### Statistical analysis

The patient characteristics as well as the dimensions of the bleb(s) of the BGI complex were compared between the patients with and without diplopia. All variables were tested separately for significance. P-values <0.05 were considered as statistically significant. For data with a normal distribution, we used the unpaired t-test (age, IOP) or Fisher’s Exact test (number of patients per group). For data with a non-normal distribution, we used the Mann-Whitney test (visual field, bleb volume, bleb height). Correlations were determined by using Spearman correlation.

## Results

### Patient characteristics

In total, 30 eyes of 30 patients were measured and analyzed (Table 1). The patients were divided in two groups, one with patients that experienced diplopia (n = 12) and another with those that did not experience diplopia (n = 18). There were no significant differences between the demographic characteristics of the two groups, except for the mean deviation (MD) of the visual field in the studied eye and the presence of restricted ocular ductions (p = 0.011). The patients without diplopia had a larger average visual field loss in the studied eye (p = 0.018) and less restricted ocular movements (p = 0.011) than the patients with diplopia. The Ocular restrictions occurred mostly in adduction (50% of the patients), depression (43% of the patients) and abduction (37% of the patients). When restrictions were present, they were the largest in depression, abduction and adduction.

**Table 1:** Patient characteristics at baseline.

Characteristics	All patients n = 30	Patients with diplopia n = 12	Patients without diplopia n = 18	p-value
**Age, mean ± SD [years]**	67.3 ± 8.0	66.5 ± 9.0	67.8 ± 7.4	0.06*
**Study eye, n (%)** OD OS	16 (53%) 14 (47%)	4 (33%) 8 (67%)	12 (67%) 6 (33%)	0.13^†^
**Gender, n (%)** Men Women	17 (56%) 13 (44%)	7 (58%) 5 (42%)	10 (56%) 8 (44%)	1.0^†^
**IOP, mean ± SD [mmHg]** Preoperatively Postoperatively 6 weeks Postoperatively 3 months Postoperatively at MRI visit	21.0 ± 5.9 16.4 ± 7.8 16.2 ± 6.2 12.0 ± 3.1	21.9 ± 7.3 17.7 ± 8.1 17.1 ± 5.3 13.4 ± 4.0	20.4 ± 5.0 15.5 ± 7.7 16.8 ± 6.7 11.3 ± 2.3	0.60^‡^ 0.37^‡^ 0.55^‡^ 0.18^‡^
**Surgical technique, n (%)** Free plate Sutured plate	15 (50%) 15 (50%)	7 (58%) 5 (42%)	8 (44%) 10 (56%)	0.71^†^
**End plate position, n (%)** Superotemporally Superonasally	29 (97%) 1 (3%)	12 (100%) 0	17 (94%) 1 (6%)	1.0^†^
**Time after surgery, mean ± SD (range) [years]**	4.7 ± 2.5 (1.5–10)	3.6 ± 1.4 (2–5)	5.3 ± 2.9 (1.5–10)	0.12^‡^
**Visual Field MD, median (IQR; Q3-Q1)** study eye Fellow eye	-9.0 (-6.8-(-13.5)) -3.3 (-1.5-(-6.1))	-6.9 (-2.2-(9.9)) -3.0 (-0.2-(-6.1))	-12.17 (-8.9-(-15.2)) -3.5 (-2.2-(-6.2))	**0.017**^‡^ 0.46^‡^
**Restricted ocular ductions, n (%)**	16 (53%)	10 (83%)	6 (33%)	**0.01** ^†^

SD = standard deviation; OD = right eye; OS = left eye; IOP = intraocular pressure; MD = mean deviation; IQR = interquartile range

Study eye = eye with BGI; fellow eye = eye without BGI

* unpaired t-test

^†^ Fisher Exact test

^‡^ Mann-Whitney test

### Magnetic resonance imaging of the BGI

**Fig 2:**
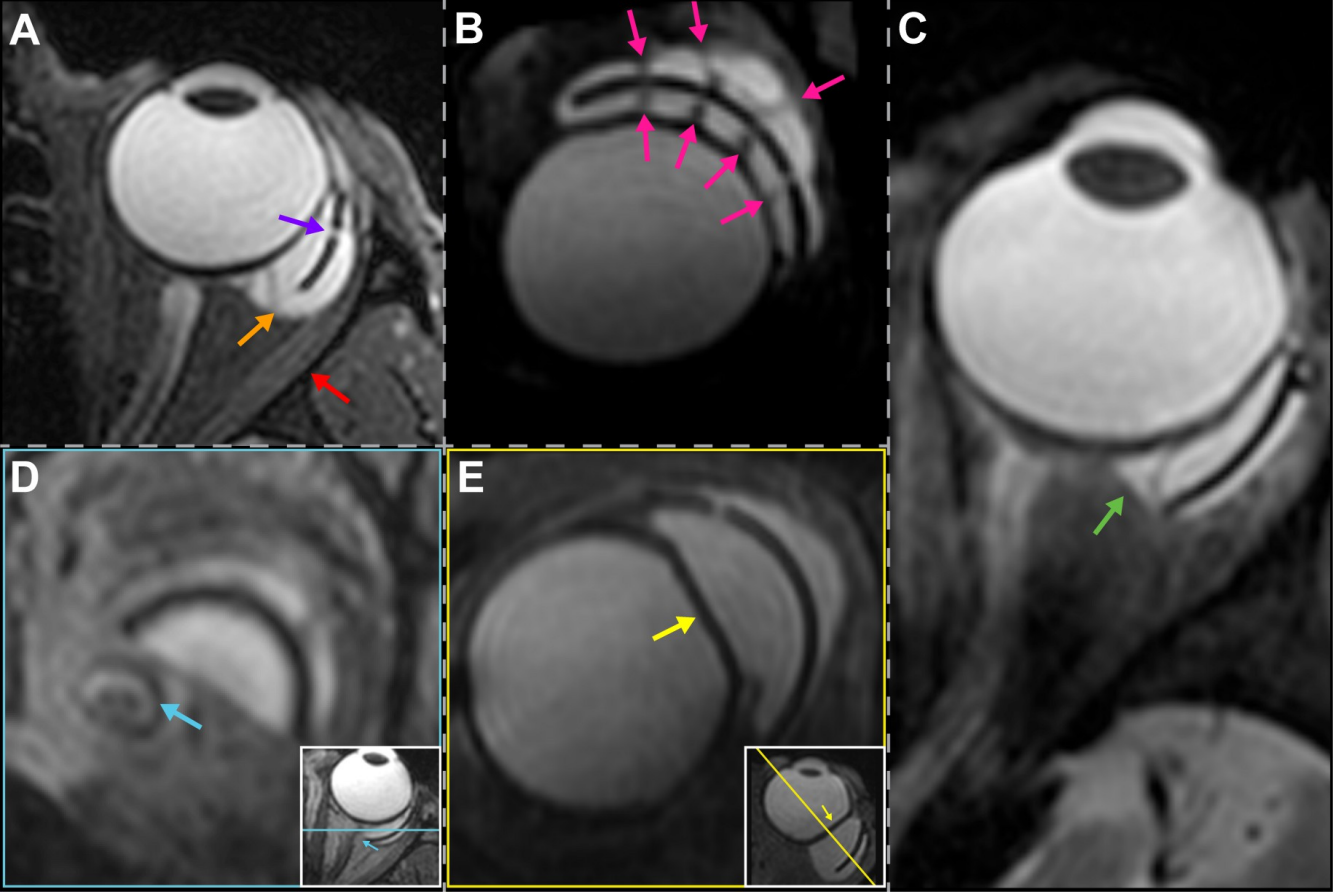
Overview of findings on MR-images-of patients with a BGI. A) Subject with a relatively large inner and outer bleb that seem to touch, resulting in one large bleb surrounding the BGI plate (*orange arrow*). Additionally, a fenestration in the plate *(purple arrow)* is visible. Moreover, the lateral rectus muscle *(red arrow)* appears to be pushed away from the eye by the BGI complex. B) Thin fiber-like hypo-intense structures running through the bleb(s) and the plate fenestrations *(pink arrows)*. C) 2D MR-images showing a small amount of extra fluid located directly outside of the original blebs *(green arrow)*. D) Contact between the BGI complex and the optic nerve *(blue arrow)*. E) Deformation of the globe due to the presence of the BGI complex *(yellow arrow)*.

On the 3D T2-weighted MR-images, the silicone plate was surrounded by one or two blebs (Fig 2) and could be clearly identified. In 24 patients (80%), an inner bleb (i.e., between the globe and the plate) and an outer bleb (i.e., on top of the plate) could be distinguished. However, in 6 patients (20%), the blebs were large enough to touch each other at the edges, in which case it seemed as though the plate was floating inside a large bleb (Fig 2A). The plate appeared hypo-intense on T2-weighted images, whereas the blebs were iso-intense to the vitreous on both T1 and T2 weighted images. On these images, the bleb-sclera interface was generally visible, and the small fenestrations of the BGI’s plate could be identified in most of the subjects (Fig 2A). Furthermore, on T2 thin sheet- or fiber-like hypointense structures were visible in both the inner and outer bleb(s) in 26 out of 30 patients (87%) (Fig 2B). These structures run through the fenestrations of the BGI’s plate, suggesting they form a continuous structure from the inner to the outer bleb.

The 3D MR-images allowed a more accurate assessment of the bleb volume than 2D cross-sections, especially for the larger blebs whose highly asymmetric geometry could easily be missed on 2D cross-sections. Furthermore, the 3D images enabled a 3D evaluation of the relationship between the blebs and surrounding structures as the images could be reformatted in any direction without loss of image quality. In 9 patients, we observed a small hyperintense region on T2 directly adjacent to the bleb and outside of the capsule (Fig 2C), which is likely fluid filling the space between the bleb and sclera. In one of the patients, the bleb was in direct contact with the optic nerve, resulting in a medial shift of the optic nerve (Fig 2D). This patient had advanced visual field loss (MD of study eye = -13 dB). Finally, in 6 patients, a deformation of the globe due to the presence of the bleb(s) was observed (Fig 2E). No significant difference was found between the IOP or VF loss of the patients with and without thisdeformation (p = 0.67; p = 0.51; Mann-Whitney test).

### Bleb size versus diplopia

**Fig 3:**
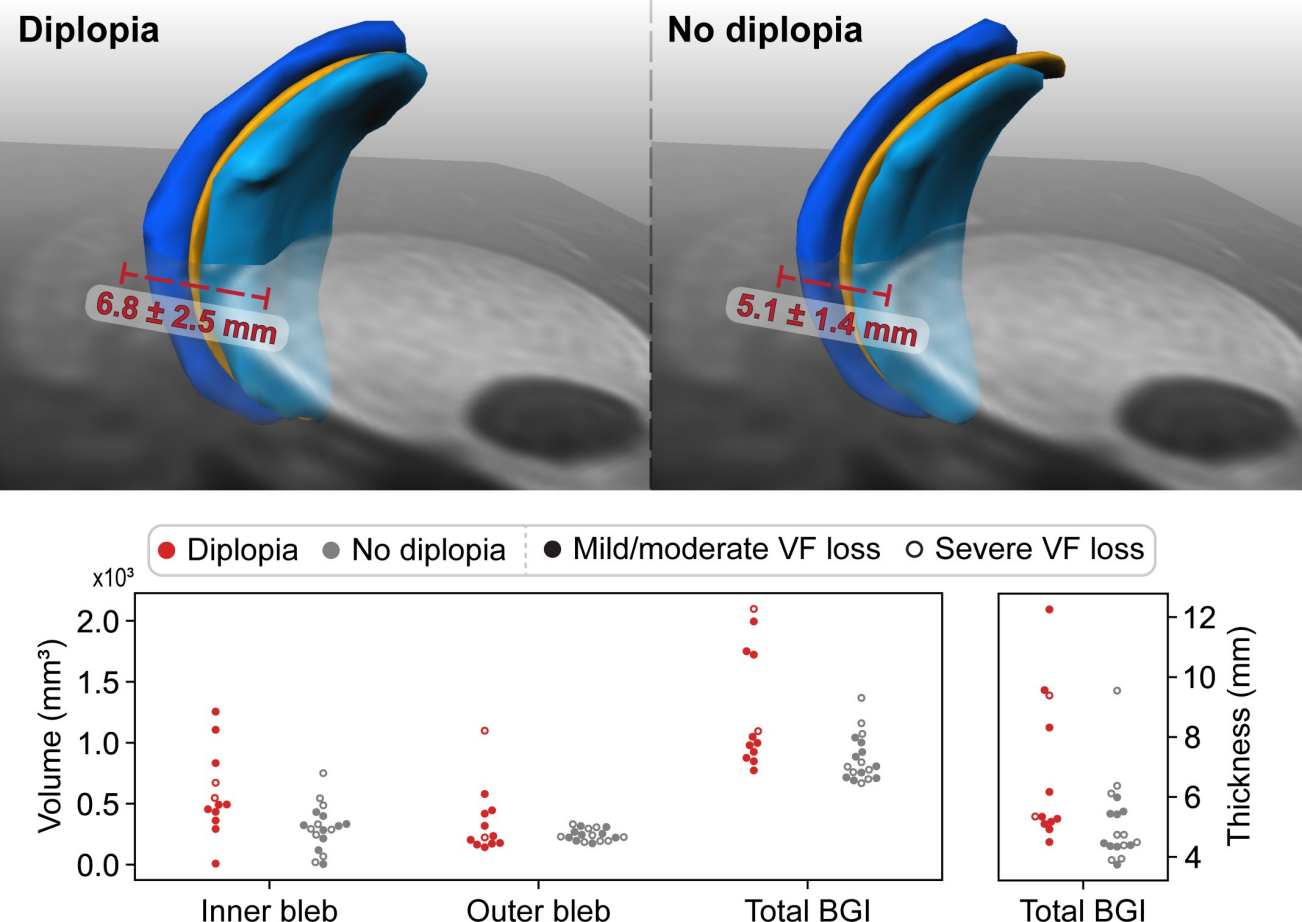
Comparison between the BGI of patients with and without diplopia. *Top left)* The average BGI complex volume for patients with diplopia, with the average largest thickness in red. *Top right)* The average BGI complex volume for patients without diplopia, with the average largest thickness in red. *Bottom)* Scatterplots of the individual BGI dimensions of patients (each dot represents a patient) with *(red)* and without *(grey)* diplopia. The filled or not open dots represent the visual field loss category of the patient with a filled dot being a patient with mild to moderate visual field (VF) loss (MD>-12dB), and an open dot being a patient with advanced visual field loss (MD<-12dB).

On average, the volume of the BGI complex was significantly larger in patients with diplopia than in patients without diplopia (1023.3mm^3^ vs 804.6mm^3^, Table 2; Fig 3). Additionally, the overall height of the BGI complex, measured perpendicular to the plate, was larger in the diplopia group compared to the asymptomatic group (Table 2). A significant correlation was found between experiencing diplopia and the total volume as well as the overall height of the BGI complex (p = 0.005 and p = 0.022; r_s_ = 0.495 and r_s_ = 0.417 respectively; Spearman correlation). Additionally, we found a correlation between the total amount of ocular motility restriction (all gaze directions considered) and the total volume of the BGI complex (p = 0.041; r_s_ = -0.374; Spearman correlation). The more the eye movement was restricted, the larger the BGI complex volume was.

**Table 2:** Measured bleb sizes for each patient group.

	Patients with diplopia n = 12	Patients without diplopia n = 18	p-value*
**BGI complex volume (plate included), median (IQR) [mm** ^**3**^ **]**	1023.3 (875.7–1750.5)	804.6 (755.1–923.4)	**0.007**
**Outer bleb volume, median (IQR) [mm** ^**3**^ **] (i.e. bleb on top of the plate)**	228.6 (171.9–445.1)	234.8 (221.5–268.3)	0.917
**Inner bleb volume, median (IQR) [mm** ^**3**^ **] (i.e. bleb between the globe and the plate)**	492.3 (361.5–832.7)	304.9 (244.8–333.2)	**0.006**
**BGI complex thickness (plate included), median (IQR) [mm]**	5.3 (5.1–9.3)	4.6 (4.4–5.4)	**0.025**

IQR = interquartile range

* Mann-Whitney test

In total, 6 patients had an excessively large total bleb volume (>1100 mm^3^) (median of 1736.5 mm^3^, IQR 1486.3–1933.9 mm^3^), of which 4 experienced diplopia (33% of the patients with diplopia) and had motility restrictions. The 2 patients without diplopia but with a large bleb volume showed motility restrictions and severe visual field loss in the study eye (MD < -12 dB) (Fig 3).

Of the 6 patients on which a deformation of the globe was observed on the MR-images, 4 had a large bleb volume. Of these 4 patients, all experienced diplopia and 3 showed signs of proptosis. The bleb of the patient in whom the bleb was in direct contact with the optic nerve, had a normal volume.

Neither the total bleb volume nor the bleb height was correlated with the IOP; total bleb volume compared to IOP preoperatively, IOP at 6 weeks and 3 months post-operatively and during the last visit were p = 0.35, p = 0.92, p = 0.96 and p = 0.86, respectively, while the bleb height compared to IOP preoperatively, IOP at 6 weeks and 3 months post-operatively and during the last visit were p = 0.06, p = 0.96, p = 0.88 and p = 0.93, respectively (Spearman correlation).

Lastly, in most patients we were able to visualize fibrotic strands (Fig 2B), regardless of the presence of any diplopia or not (75% versus 88% respectively; p = 0.278; Fisher Exact test), while the extra fluid next to the bleb (Fig 2C) was more common among patients without diplopia than among patients with diplopia, 44% versus 8% respectively; p = 0.049).

## Discussion

By using magnetic resonance imaging, we have shown for the first time that a large bleb in the orbit can restrict the ocular movement, which probably explains the diplopia in some of the patients. One out of 3 of our patients with diplopia seemed to have developed an excessively large bleb.

We identified 3 studies that have characterized and quantified bleb sizes 6 months or more after glaucoma drainage device surgery, by means of magnetic resonance imaging. In all 3 studies, the bleb volumes were smaller than in our results. Iwasaki et al [16], who included 52 eyes with a BGI, detected a total bleb volume of 230 ± 150 mm^3^ (mean ± SD). At 6 months postoperatively, Sano et al. [15] analyzed bleb images of 19 eyes with a BGI and found a bleb volume of 478.8 ± 84.2 mm^3^. Detorakis et al. [14] found a bleb volume of 240 ± 100 mm^3^ in patient with the Ahmed implant and 180 ± 140 mm^3^ in patients with a Molteno implant. All 3 studies calculated the volumes of the blebs without the volume of the plate. We chose to include both the plate height and volume in our analyses because they provide this corresponds to the total orbital space that is occupied by the BGI complex, which might lead to restrictions in ocular movement. In addition, all 3 studies involved Asian subjects. Asians tend to have a taller, more-circularly shaped orbit, whereas Caucasians tend to have a square-shaped orbit [24]. This difference in orbital anatomy may result in different bleb volume development. It would be valuable to assess the relation between bleb, orbital volume and axial length, which can both be obtained with MRI [25], and the orthoptic measures, to gain a better understanding of when the mass effect of a large bleb induces diplopia. Additionally the potential relation between extra-ocular muscle insertions and the position of the BGI in patients with diplopia warrants further study.

In addition, Sano et al. [15] pointed out that patients with a BGI had a significantly larger bleb volume compared to patients with an Ahmed implantation. Importantly, none of these studies correlated the bleb size with the presence of any diplopia.

Similar to the results of Iwasaki et al. [16] and Sano et al. [15], we did not find a correlation between postoperative IOPs and bleb volume. Clinically, bleb formation i.e., the encapsulation of the plate, takes place within the first 6 weeks postoperatively, so that, by the time the resorbable ligature around the tube dissolves, the aqueous may flow from the anterior chamber into the bleb to lower the IOP.

It appears that a bleb can become so large that it compresses the surrounding structures. The clinical effect of the observed deformation of the globe and optic nerve is subject to future research.

Our images demonstrated that most bleb structures have four fibrotic strands, which correspond to the four fenestrations of the BGI plate. Iwasaki et al. [16] have indicated the visibility of four holes on top of the bleb (not the plate) on MRI. However, fibrotic strands inside the bleb have not been identified on images before. The fenestrations in the BGI plate were created by the manufacturer to allow the development of fibrous strands between the sclera and the bleb roof through the plate [26]. The aim of these fibrotic strands is to flatten the bleb and thereby restrict the bleb size. It is believed that by preventing the formation of oversized blebs, the incidence of diplopia is reduced [27]. Unfortunately, our results suggest that this design is—in some cases—not sufficient to restrict the bleb size and can therefore not prevent diplopia in all patients. We should point out that the visibility of these strands depends highly on the image quality and contrast of the MR-images. The used cued-blinking paradigm [22] technique was highly efficient in reducing eye-motion artefacts, which would otherwise render these thin structures invisible due to motion blurring. Moreover, optimizing the contrast generated by the MR-sequence is crucial to identify these subtle differences between various intraorbital structures.

Our study, unfortunately, was not longitudinal by design. Therefore, we could not study the chain of events in implant encapsulation, bleb formation, scarring and the formation of any adhesions between the bleb capsule and any other intraorbital structures. Such information might help preventing the development of oversized blebs and should be investigated in future studies. Obtaining several MR-images at different time points during the first postoperative months could provide more information in the development of the blebs.

Not all cases of diplopia could be explained by the presence of a large bleb. In patients with diplopia but no large bleb volume, the diplopia may have been caused by a different mechanism such as the development of fibrous adhesions between the outside of the capsule and adjacent tissues. The MR images did not show any clear development of tight fibrous adhesions between the bleb capsule and the surrounding tissues. However, as the used sequences were primarily designed for an accurate geometric assessment of the BGI complex and its relation to the surrounding structures, the resulting images probably did not have the correct contrast to detect such small fibrous layers. In clinical practice, though, we know that in some of our patients in whom the severity of their diplopia led to surgical revision of the BGI, tight fibrous adhesions between the capsule and the surrounding tissues, including the recti muscles, were found. Future studies employing quantitative, MR-imaging methods, such as T_1rho_ mapping [28], quantifying fibrotic tissue changes, and T_2_ mapping [29], a biomarker for muscle inflammation and early predictor of muscle damage, could aid to gain insight in these potential effects of the BGI on the recti muscles. Furthermore, in one of the patients a clear mass effect of the BGI complex was observed where the BGI complex displaced the lateral rectus muscle, providing an alternative cause of diplopia (Fig 2A). The future inclusion of a dedicated MRI scan visualizing the BGI-muscle relationship could clarify if a similar effect could be present in diplopic patients with a smaller BGI complex. Dynamic MR-imaging, in which the subject is asked to gaze into different directions, could provide additional clues on the gaze dependency of diplopia. For such a study a different MRI setup is however required, as the used head coil obstructs the view for larger gazing angles. More recent ocular MRI protocols, however, do provide sufficient space for fixation targets at high eccentricities [30].From a clinical perspective, the ophthalmologist should consider that a large bleb size may be at the origin of diplopia following BGI implantation. By contrast, when a patient does not report diplopia it does not always mean that eye motility has not been affected. Visual field loss has an effect on whether patients experience diplopia or not [8]. Based on their large bleb volumes, we would have expected that 2 patients in our study should experience diplopia although they did not report such symptoms. We speculate that their lack of symptoms was probably due to severe visual field loss.

Diplopia is a serious complication that may have a negative impact on the patient’s psychosocial well-being [31, 32]. A possible solution for patients with persistent diplopia and a large bleb can be a surgical revision in which a large bleb is emptied by the surgeon, effectively reducing the bleb size. Subsequently, the surgeon may temporarily ligate the tube again to prevent the aqueous from draining so that the bleb may settle to a lower volume. Such a procedure has been effective in some of our patients.

In conclusion, a large bleb can be at the origin of the development of diplopia after BGI surgery but is not the only possible cause for diplopia. Fibrotic adhesions between the bleb capsule and its surrounding tissues could also play a role in the diplopia. More research is necessary, especially for investigating such fibrotic adhesions. MR-images obtained at several time points, with additional scans aiming to visualize fibrotic adhesions, may help identifying anatomical changes following BGI implantation.

## Supporting information

S1 Data (XLSX)
